# Extensively drug-resistant *Acinetobacter baumannii*: role of conjugative plasmids in transferring resistance

**DOI:** 10.7717/peerj.14709

**Published:** 2023-01-25

**Authors:** Qutaiba Ababneh, Sara Al Sbei, Ziad Jaradat, Sebawe Syaj, Neda’a Aldaken, Hamza Ababneh, Zeina Inaya

**Affiliations:** 1 Department of Biotechnology and Genetic Engineering, Faculty of Science and Arts, Jordan University of Science and Technology, Irbid, Jordan; 2 Department of General Surgery and Urology, Faculty of Medicine, Jordan University of Science and Technology, Irbid, Jordan; 3 Faculty of Pharmacy, Jordan University of Science and Technology, Irbid, Jordan

**Keywords:** *Acinetobacter baumannii*, Whole genome analysis, Extensively drug resistance, Conjugative plasmids

## Abstract

*Acinetobacter baumannii* is one of the most successful pathogens that can cause difficult-to-treat nosocomial infections. Outbreaks and infections caused by multi-drug resistant *A. baumannii* are prevalent worldwide, with only a few antibiotics are currently available for treatments. Plasmids represent an ideal vehicle for acquiring and transferring resistance genes in *A. baumannii*. Five extensively drug-resistant *A. baumannii* clinical isolates from three major Jordanian hospitals were fully sequenced. Whole-Genome Sequences (WGS) were used to study the antimicrobial resistance and virulence genes, sequence types, and phylogenetic relationship of the isolates. Plasmids were characterized *In-silico*, followed by conjugation, and plasmid curing experiments. Eight plasmids were recovered; resistance plasmids carrying either aminoglycosides or sulfonamide genes were detected. Chromosomal resistance genes included *bla_OXA-66_, bla_OXA-91_, and bla_OXA-23,_*and the detected virulence factors were involved in biofilm formation, adhesion, and many other mechanisms. Conjugation and plasmid curing experiments resulted in the transfer or loss of several resistance phenotypes. Plasmid profiling along with phylogenetic analyses revealed high similarities between two *A. baumannii* isolates recovered from two different intensive care units (ICU). The high similarities between the isolates of the study, especially the two ICU isolates, suggest that there is a common *A. baumannii* strain prevailing in different ICU wards in Jordanian hospitals. Three resistance genes were plasmid-borne, and the transfer of the resistance phenotype emphasizes the role and importance of conjugative plasmids in spreading resistance among *A. baumannii* clinical strains.

## Introduction

*Acinetobacter baumannii* is a human opportunistic pathogen responsible for community and nosocomial-acquired infections, especially in immunosuppressed patients who experience prolonged hospital stays. Among *Acinetobacter* spp., *A. baumannii* is the main causative agent of nosocomial infections, accounting for 2% of the nosocomial infections in Europe and the United States (Magill et al., 2014; Lob et al., 2016). This pathogen has a tremendous clinical impact as it can cause life threatening infections such as bacteremia and ventilator-associated pneumonia (VAP) (Shirmohammadlou et al., 2018). In this light, the World Health Organization (WHO) has classified *A. baumannii* as a top priority for research and development of new antibiotics (Centers of Disease Control and Prevention (CDC), 2019).

*A. baumannii* has an exceptional ability to acquire multi-, extensive-, and pan-drug resistance phenotypes, and currently, more than 50% of the worldwide *A. baumannii* clinical isolates are carbapenem-resistant (Howard et al., 2012; Piperaki et al., 2019). The plasticity of the *A. baumannii* genome has a crucial role in resistance development. This bacterium can upregulate its intrinsic resistance mechanisms and acquire new resistant determinants by horizontal gene transfer (Harding, Hennon & Feldman, 2018; Rolain et al., 2013). Plasmids represent a robust vehicle for acquiring and transferring resistance genes in *A. baumannii*. Several studies have reported plasmid-mediated resistance. In addition, bacterial mating assays showed the ability of some plasmids to transfer resistance to other *A. baumannii* and even to other bacterial species (Salgado-Camargo et al., 2020; Krahn et al., 2016)*.* For example, plasmids harboring different genes encoding *β*-lactamases such as *bla*_*OXA*−72_*, bla*_*OXA*−51−__like__,_ *and bla*_*OXA*−23_ have been widely reported (Kuo et al., 2013; Chen et al., 2010; Hamidian et al., 2014b). Further, the gene encoding the New Delhi Metallo- *β*-lactamase (NDM) enzyme was also found to be carried on a plasmid in more than one *A. baumannii* isolates, and the detected plasmid-mediated gene *bla*_*NDM*−1_ was surrounded by *ISAba125* upstream and *ISAba14* downstream (Jones et al., 2014; Salloum et al., 2018). A diverse group of *A. baumannii* plasmids have been described and were found to be distributed with a closely related backbone in different isolates around the world (Lean & Yeo, 2017; Salgado-Camargo et al., 2020). However, among different *A. baumannii* plasmid groups, plasmids of group 6 (GR6) were the most prevalent in carbapenem-resistant *A. baumannii* and they showed the ability to transfer many resistance genes like *bla*_*OXA*−23_ and *aphA6*. (Towner et al., 2011; Leungtongkam et al., 2018)

New sequencing technologies and bioinformatics tools have widely contributed to identifying antimicrobial resistance genes and tracking resistance exchange networks. Indeed, detecting resistance determinants and resistance plasmids from Whole-Genome Sequences (WGSs) provided unparalleled insights into understanding the spread of drug-resistant bacteria (Boolchandani, D’Souza & Dantas, 2019). In this study, we characterized the resistome and virulome of extensively drug-resistant *A. baumannii* clinical isolates from Jordan, focusing on plasmids implicated in the transmission of antibiotic resistance. Also, sequence typing, phylogenetic analysis, plasmid curing, and conjugation experiments were carried out to gain information about the evolutionary and molecular characteristics of these clinical isolates.

## Materials & Methods

### Bacterial Isolates and *A. baumannii* Identification

The five XDR *A. baumannii* clinical isolates included in the study were recovered from patients admitted to Jordanian hospitals in 2018. Two of these isolates were recovered from patients admitted to the ICUs; one isolated (ICU29) from sputum samples of an 84-year-old female, while the other (ICU40) was isolated from the cerebrospinal fluid sample of a 5-week-old male baby. The other three non-ICU isolates were recovered from a sputum sample of a 65-year-old male (Ab19), a urine sample of a 16-year male (Ab40), and a sputum sample of a 63-year female (Ab119). The isolates were identified biochemically using the VITEK©2. Molecular identification of *A. baumannii* was carried out by PCR amplification of internal fragments of the *bla*_*OXA*−51_ gene (ying et al., 2015). This study was approved by the Jordanian Ministry of Health and the Institutional Review Board under the number MOH REC 180030.

### Antibiotic susceptibility testing and minimal inhibitory concentration (MIC)

The antibiotic susceptibility testing was performed according to the Clinical and Laboratory Standards Institute (CLSI) guidelines following the method described previously (CLSI-2021) (Hudzicki, 2009). The antibiotics used in this study and their concentrations are listed in Table S1. MICs were determined for tigecycline, colistin, imipenem, and polymyxin B (SPL Life Science) drugs using the 96-well microdilution method (Wieg, Hilpert & Hancock, 2008). Polysorbate was added for colistin sulfate and polymyxin B to improve the delivery of antibiotics at a concentration of 0.002% in each well (Sader et al., 2012).

### Next-generation sequencing and assembly

Whole-genome sequencing of the five isolates was done using two different sequencing platforms; isolate Ab119 was sequenced using Illumina and PacBio platforms. The other four isolates were sequenced using the Illumina platform only. DNA was extracted using Wizard genomic DNA purification kit (Promega, USA), and library preparation was done using the TruSeq Nano DNA Kit (Illumina) following the manufacturer’s instructions. The obtained 150bp short paired-end and long reads were assembled by SPAdes (version 3.14.0), and the genome of *A. baumannii* ATCC 19606 (NZ_CP045110) was used as a reference for the assembly (Bankevich et al., 2012). The quality of assembly was tested using the Sequencing Quality Assessment Tool (SQUAT) with the default settings for both (Yang et al., 2019).

### Annotation, sequence typing, and phylogenetic classification

Assembled contigs were annotated using Prokka and the Rapid Annotation using Subsystem Technology (RAST) servers (Seemann, 2014; Aziz et al., 2008). The sequence types (ST) were determined as described by PubMLST (https://pubmlst.org) using the locus schemes Oxford and Pasteur separately and then order results by locus (Jolley, Bray & Maiden, 2018). Phylogenetic analysis was performed using RAxML-VI-HPC (randomized accelerated maximum likelihood for high-performance computing) tool provided by EDGE Bioinformatics (https://edgebioinformatics.org) to compare the five isolates against the *A. baumannii* database, and the Average Nucleotide Identity (ANI) was calculated as described previously (Philipson et al., 2017; Stamatakis, 2006; Goris et al., 2007).

### Detection of antimicrobial resistance and virulence genes

Antimicrobial resistance genes were detected using ResFinder and Resistance Gene Identifier (CARD RGI) (https://card.mcmaster.ca). In the CARD RGI, only perfect and strict hits were chosen, while loose hits with less than 95% identity were excluded (Zankari et al., 2012; Alcock et al., 2020). Virulence genes were detected using the virulence factor database (VFDB) through the EDGE bioinformatics platform (Philipson et al., 2017; Chen et al., 2005).

### Plasmid identification

*In silico* identification of plasmids was initially done using plasmidSPAdes and then confirmed using BlastN at NCBI (Antipov et al., 2016). PlasmidSeeker subsequently analyzed confirmed plasmid contigs to find the closest reference plasmid (Roosaare et al., 2018). RAST annotated the verified plasmids, and manual annotation for the replication (rep) genes was based on Bertini classification (Bertini et al., 2010).

### Conjugation assay

Azide-resistance induction in *A. baumannii* recipient strains was performed as described previously (Leungtongkam et al., 2018)**.** The donor and recipient cells were mixed at a 1:3 ratio in Luria-Bertini broth and incubated at 37 °C for 4 h. The selection was made on Mueller Hinton Agar supplemented with 300 µg/ml sodium azide and the appropriate antibiotic. Transconjugants were collected and tested against several antibiotics to detect the transfer of resistance genes by conjugation (Leungtongkam et al., 2018).

### Plasmid curing

The curing agent acridine orange was used for plasmid curing as described previously (Saranathan et al., 2014). The curing broth was prepared by diluting acridine orange to the subinhibitory concentration using LB broth to a final volume of 10 ml. The tested isolates were inoculated into the curing broth, incubated for 48 h, and plated on LB agar. The antibiotic susceptibility testing was done for the isolates before and after curing to compare their antibiotic resistance profiles (Trevors, 1986).

### Nucleotide sequences accession number

The complete sequence of the five isolates are deposited under Bio project number PRJNA739752. The five assembled genomes Ab40, Ab19, Ab119, ICU40, and ICU29 are assigned accession numbers GCA_019891135.1, GCA_019891055.1, GCA_019891085.1, GCA_019891095.1, and GCA_019891065.1, respectively.

## Results

### Antimicrobial resistance

The five *A. baumannii* isolates investigated in this study were classified as XDR based on their antimicrobial resistance profiles (Table 1). Four isolates exhibited resistance against 18 out of the 21 tested antibiotics, but all were sensitive to tigecycline, polymyxin B, and colistin sulphate. Isolate Ab40 was susceptibility to trimethoprim-sulfamethoxazole and tobramycin and showed intermediate susceptibly to gentamicin. Additionally, isolate ICU40 showed intermediate susceptibility to trimethoprim-sulfamethoxazole.

**Table 1:** Resistance profile of the isolates: results generated from antibiotic susceptibility test and MIC.

**Tested antibiotic**	**Ab19**	**Ab40**	**Ab119**	**ICU29**	**ICU40**
Tigecycline	–	–	–	–	–
Tetracycline	+	+	+	+	+
Trimethoprim-Sulphamethoxazole	+	–	+	+	-/+
Piperacillin/Tazobactam	+	+	+	+	+
Ampicillin-Sulbactam	+	+	+	+	+
Ampicillin	+	+	+	+	+
Ceftriaxone	+	+	+	+	+
Ceftazidime	+	+	+	+	+
Cefepime	+	+	+	+	+
Ertapenem	+	+	+	+	+
Doripenem	+	+	+	+	+
Imipenem	+	+	+	+	+
Meropenem	+	+	+	+	+
Ciprofloxacin	+	+	+	+	+
Levofloxacin	+	+	+	+	+
Norfloxacin	+	+	+	+	+
Gentamicin	+	-/+	+	+	+
Amikacin	+	+	+	+	+
Tobramycin	+	–	+	+	+
Colistin Sulphate	–	–	–	–	–
Imipenem (10 mcg) MIC value (ug/ml)	4 ug/ml (-/+)	4 ug/ml (-/+)	8 ug/ml (+)	8 ug/ml (+)	32 ug/ml (+)
Tigecycline (15 mcg) MIC value (ug/ml)	1 ug/ml (-)	1 ug/ml (-)	1 ug/ml (-)	2 ug/ml (-)	2 ug/ml (-)
Colistin (10 mcg) MIC value (ug/ml)	1 ug/ml (-)	0.5 ug/ml (-)	0.5 ug/ml (-)	0.5 ug/ml (-)	2 ug/ml (-)
Polymyxin B (10 mcg) MIC value (ug/ml)	1 ug/ml (-)	1 ug/ml (-)	0.5 ug/ml (-)	1 ug/ml (-)	0.5 ug/ml (-)

**Notes.**

+ Resistant - Susceptible -/+ Intermediate resistance

**Table 2:** Multiple locus sequence typing (MLST) of the isolates according to both the Pasteur Institute and Oxford schemes.

Isolates	Ab19	Ab40	ICU29	ICU40	Ab119
ST (Pasteur scheme)	2	164	600	600	ND
ST (Oxford schemes)	1114, 452	1418	1305, 2026	1632	New ST
Clonal Complex (CC)	2	40	2	2	–

### Whole genome sequencing and sequence types

The reference-based assembly of the five Illumina sequenced isolates yielded an average length of 4,520,996 bp and an average G+C content of 38.76%. The number of detected open reading frames (ORFs) ranged between 3,754 and 4,324, with half of the ORFs coding for hypothetical proteins. Details of the WGSs results are listed in Table S2. Multiple locus sequence typing (MLST) showed that isolate Ab119 has a new ST according to the Oxford scheme and was not identified by the Pasteur scheme (Table 2). Details of MLST analysis can be found in Tables S3 and S4.

### Antimicrobial resistance determinants

The isolates harbored an average of 27 different genes and gene mutations conferring resistance to antimicrobial agents through various mechanisms (Table 3). Isolate ICU40 contained the highest number of resistance determinants, whereas isolate Ab119 had the least. Genes that encode the resistance-nodulation-cell division (RND) efflux pumps were detected in all isolates. Tetracycline resistance MFS efflux pumps were less frequent; *tetR* and *tetB* were only found in isolates Ab19, ICU29, and ICU40. On the other hand, *tet39* was only found in isolates ICU40 and Ab40. Two types of *bla*_*OXA*_ genes, *bla*_*OXA*−66_ and *bla*_*OXA*−23_, were found in most isolates, while *bla*_*OXA*−91_ was only found in Ab40. These genes encode OXA-type beta-lactamases, conferring resistance to carbapenems, cephalosporins, and penams. Other carbapenem resistance genes detected include *bla*_*ADC*−30_*, bla*_*ADC*−25_*, bla*_*ADC*−81_, and *bla*_*ADC*−73_. The *bla*_*NDM*−1_ gene encoding NDM-1 (zinc Metallo- *β*-lactamase “MBL”) was found in ICU40. In addition, the Extended-Spectrum *β*-Lactamase (ESBL) gene *bla*_*TEM*−1_ was detected in three isolates. Aminoglycoside resistance genes were found in all isolates, with *ant(2′)Ia*, *aph(3′)VI*, and *aph(3′)VIa* being plasmid-borne. Two mutations in the fluoroquinolone-resistance determining regions (QRDRs) of *gyrA* and *parC* genes were detected in all isolates. Isolate Ab19 carries the disinfectant-resistance *qacE/qacE* Δ*1* gene.

**Table 3:** Antimicrobial Resistance genes classified according to the mechanism of action. AMR genes were detected using CADR RGI and ResFinder. The positive sign (+) indicates the detection of the gene, and the plasmid-borne genes are indicated by the (+p).

***Gene name***		**Ab19**	**Ab40**	**Ab119**	**ICU29**	**ICU40**		***Antimicrobial family***
*adeI and adeK (RND)*		**+**						Multidrug
*adeA, adeC, adeG (RND)*		**+**		**+**				Multidrug
*adeL, adeR, adeF (RND)*		**+**	**+**	**+**	**+**	**+**		Multidrug
*adeN (RND)*		**+**	**+**	**+**				Multidrug
*adeH (RND)*		**+**		**+**	**+**	**+**		Multidrug
*adeJ (RND)*		**+**	**+**		**+**	**+**		Multidrug
*abeS (SMR)*		**+**	**+**	**+**	**+**	**+**		Macrolide, Aminocoumarin
*abaF, amvA (MFS)*		**+**	**+**	**+**	**+**	**+**		Fosfomycin, Macrolide, fluoroquinolones
*abaQ (MFS)*		**+**	**+**		**+**	**+**		Fosfomycin, Macrolide, fluoroquinolones
*tetR (MFS)*		**+**			**+**	**+**		Tetracycline
*tetB (MFS)*		**+**			**+**	**+**		Tetracycline
*tet39 (MFS)*			**+**					Tetracycline
*Sul1*		**+**						Sulfonamides
*Sul2*		**+p**	**+**	**+**	**+**	**+**		Sulfonamides
*armA*				**+**	**+**	**+**		Aminoglycosides
*parC: S84L, V104I, D105E*		**+**	**+**	**+**	**+**	**+**		Fluoroquinolones
*gyrA: S81L*		**+**	**+**	**+**	**+**	**+**		Fluoroquinolones
*bla*_*OXA*−23_		**+**	**+**		**+**	**+**		Cephalosporin, Penams
*bla*_*OXA*−66_		**+**		**+**	**+**	**+**		Beta Lactams
*bla*_*OXA*−91_			**+**					Cephalosporin, Penams
*bla*_*ADC*−30_		**+**						Cephalosporins
*bla*_*ADC*−25_		**+**	**+**	**+**	**+**	**+**		Beta Lactams
*bla*_*ADC*−81_			**+**					Cephalosporins
*bla*_*ADC*−73_				**+**	**+**	**+**		Cephalosporins
*bla*_*NDM*−1_					**+**			Carbapenems, Cephalosporins, Cephamycin
*bla*_*TEM*−1_		**+**		**+**	**+**			Monobactam, Cephalosporins, Penams
*mphE*				**+**	**+**	**+**		Macrolide phosphotransferase
*ctx m 55*					**+**	**+**		Cephalosporin
*catb8*		**+**						Chloramphenicol
*fosa3*					**+**	**+**		Fosfomycin
*aph(3′)VIa or aph(3′)vi*		**+p**	**+**	**+**	**+p**	**+p**		Aminoglycosides
*aph(3′)Ia*								Aminoglycosides
*ant(2′)Ia*		**+p**						Aminoglycosides
*ant(3′)IIc*		**+**		**+**				Aminoglycosides
*aph(6)Id, aac(3)Ia, aac(6′)Ib9*		**+**						Aminoglycosides
*aph(3′)Ib, aph(6)Iid*		**+**	**+**		**+**	**+**		Aminoglycosides
*ant(3′) IIc*		**+**			**+**	**+**		Aminoglycosides
*aadA*		**+**						Aminoglycosides
*msrE*		**+**		**+**	**+**	**+**		Multidrug
*LpsB*		**+**	**+**	**+**	**+**	**+**		Intrinsic peptide antibiotic resistant
*qacE/qacE*Δ*1*		**+**						Quaternary ammonium compounds

### Virulence factors

An average of 60 virulence genes was detected in each isolate using the virulence factor database (VFDB). Those genes are involved in surface adherence, biofilm formation, virulence enzymes, immune evasion, iron uptake, quorum sensing, and serum resistance. Table 4 lists the virulence factors that were detected and their classification according to mechanisms of action.

**Table 4:** Virulence genes in the isolates classified according to their mechanism of action and detected by EDGE bioinformatics.

**Virulence factor mechanism of action**	**Gene name**	**Description**
**Adherence**	*ompA*	Outer membrane protein
	*pilT*	Type IV pili biosynthesis
**Biofilm formation**	*AdeH, AdeG, AdeF*	Outer membrane RND efflux pump Membrane-fusion protein
	*bap*	Biofilm-associated protein
	*csuE, D, C, B, A, csuA/B*	Csu pili
	*pgaD, C, A, B*	Polysaccharide poly-N-acetylglucosamine
**Virulence Enzyme**	*plc*	Phospholipase C
	*plcD*	Phospholipase D
**Immune evasion**	*spsC, wecE, wbbJ, mviM, wecC, pmm, ptk, ugd, weeH*	Capsule
	*lpxD, A, B, C, L, M, ipsB*	LPS
**Iron uptake**	*bauF, D, C, B, E, A, bas A, B, C, D, F, G, I, J, H, entE, barA, barB*	Acinetobactin
	*tonB, fecI*	Heme utilization
	*pvdH*	Pyoverdine
**Regulation**	*abaR, abaI*	Quorum sensing
	*bfmS, bfmR*	Two-component system
**Serum resistance**	*pbpG*	D-alanyl-D-alanine endopeptidase

### Characterization of plasmids

The computational tools detected eight plasmids; four share the same structure with some differences. The ANI between these four plasmids ranged between 99.95% and 99.76%, and thus were named pAb19.1v(1-4). Additionally, two plasmids in both ICU isolates were found identical. Out of the eight plasmids, isolate Ab19 carried 3, ICU40 had 2, and ICU29 and Ab119 each carried one plasmid (Table 5). Replication genes were identified in six of the plasmids; four “pAb19.1v(1-4)” belong to Group 6 (GR6) of *A. baumannii* plasmids and carry genes for conjugation. In contrast, pAb-ICU29 found in both ICU isolates is a small cryptic plasmid (∼9 kb) that belonged to GR2 and didn’t carry any conjugation or resistance genes. Three resistance genes were found to be plasmid-borne; *sul2*, *aph(3′)-VIa,* and *ant(2′)-Ia*.

**Figure 1:**
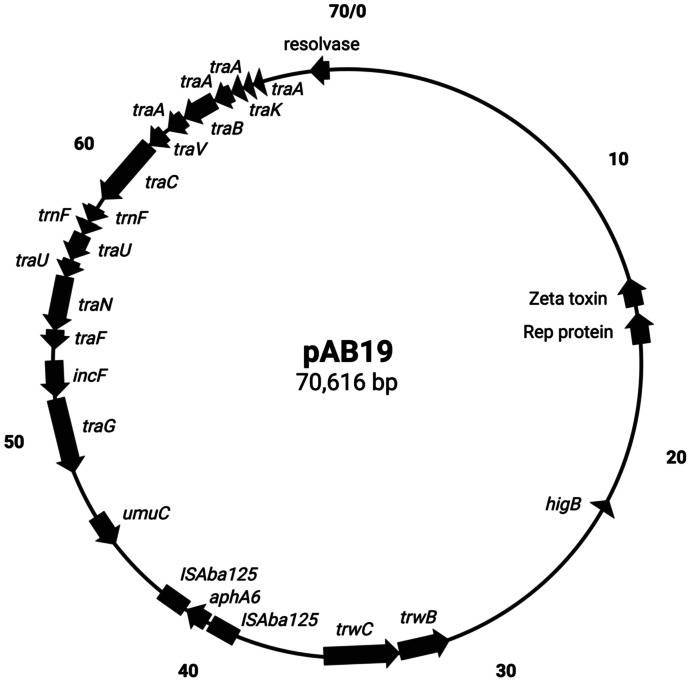
Map of conjugative pAb19.1. Figure generated by SnapGene.

**Figure 2:**
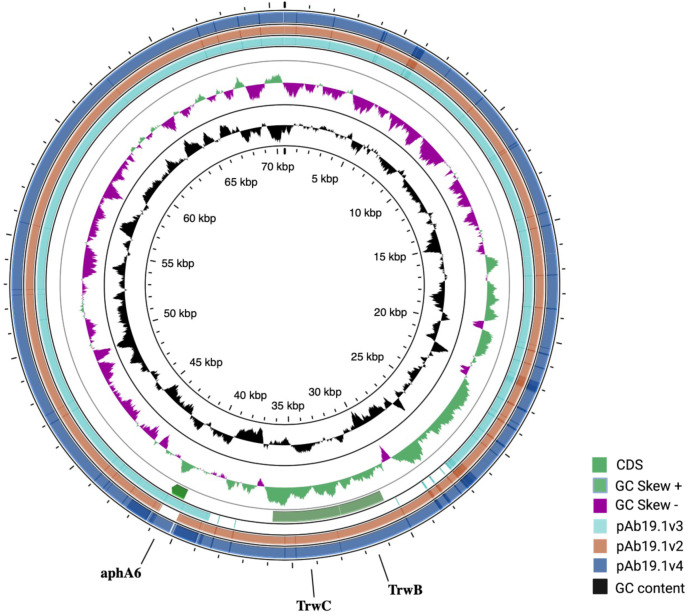
Comparing the four shared plasmids, pAb19.1, and its derivatives. The figure was generated by CGView server.

pAb19.1v1 (70,616 bp) is a large plasmid that carries the *repAci6* replication gene and belongs to GR6 of *A. baumannii* plasmids. This plasmid carried 18 conjugation genes and is highly similar to the 70,100 bp plasmid pAb-G7-2 (accession number KF669606). pAb19.1v1 has two regions of conjugation genes; type IV secretion system *tra* genes and the other region harbors the *trwB* and *trwC* mobilization genes (Fig. 1). It also carries aminoglycoside resistance transposon TnaphA6, which consists of the aminoglycoside resistance gene *aph(3′)-Via* (*aphA6*) surrounded by two IS*Aba125*. Plasmids very similar to pAb19.1v1 were detected in isolates Ab40, ICU40, and Ab119 (Fig. 2). Plasmid pAb19.1v2 (68,684 bp) from isolate Ab40 does not carry the resistance transposon TnaphA6, while pAb19.1v4 carries the transposon and is almost identical to pAb19.1v1 with a length of 70,183 bp. Although pAb19.1v3 is only 59,004 bp and does not have the second region of conjugative genes *trwB* and *trwC*, it does carry the aminoglycoside resistance transposon TnaphA6.

**Figure 3:**
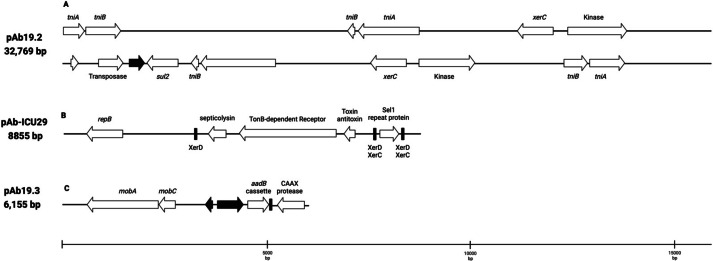
Three plasmids carrying resistance genes. (A) plasmid pAb. 2 carries *sul2* resistant gene, TniA transposase, and site-specific tyrosine recombinase XerC. (B) pAb-ICU29 carries repB, AbkB/AbkA toxin-antitoxin system, and Sel1 gene flanked by XerC/XerD recombination sites. (C) pAb19.3 carries aadB gene cassette, mobA, and mobC genes.

Plasmid pAb19.2 (32,796 bp) is harbored by Ab19 and seems a novel plasmid. Part of this plasmid is similar to plasmid pORAB01-2 (24,022 bp) from *A. baumannii* strain ORAB01 (accession number CP015485). The blast of pORAB01-2 as query and pAb19.2 as subject resulted in 99.86% percentage identity and 86% query coverage. Both plasmids carry the *E. coli*-generated sulfonamide resistance gene *sul2* surrounded by the putative transposases TniA and TniB, and other transposases. Plasmid pAb19.2 also has two site-specific tyrosine recombinase XerC and six transposases, four of which are TniA putative transposase (Fig. 3A).

Plasmid pAb19.3 (6155 bp) harbors the *aadB* gene cassette, which confers resistance to tobramycin, gentamicin, and kanamycin. The plasmid also contains *mobA* and *mobC* mobilization genes. pAb19.3 is highly similar (97.84%) to pRAY* (AF003958) (Fig. 3B). Plasmid pAb-ICU29 (8,858 bp) found in ICU29 and ICU40 isolates is almost identical to pAC29a (CP008850). However, pAC29a (8,737 bp) has two *rep* genes, *repA,* and *repB*, while pAb-ICU29 carries only the *repB* gene (Lean et al., 2016). pAb-ICU29 belongs to GR2 as it carries RepB replication protein. AbkB/AbkA toxin-antitoxin (TA), *Sel1* gene flanked by XerC/XerD recombination sites, and TonB receptor genes are encoded by both pAC29a and pAb-ICU29 (Fig. 3C).

### Induction of azide-resistant *Acinetobacter baumannii* and conjugation

Of the two environmental *A. baumannii* isolates susceptible to all tested antibiotics, one isolate (Pk1) was able to grow in the presence of 300 µg/ml sodium azide and thus was used for conjugation assay. Out of the five isolates investigated in this study, transconjugants were generated from three donors only (Ab19, Ab40, and Ab119). After conjugation, transconjugants acquired resistance against amikacin, gentamicin, cefepime, and tetracycline. Also, transconjugants became less sensitive to other antibiotics even though the phenotype was not changed into resistance (Table 6).

### Plasmid curing

The MIC of acridine orange differed between the five isolates, as Ab40, Ab119 were not able to grow on the minimum concentration (640 µg/ml) required for plasmid curing. Thus, the other three isolates (Ab19, ICU29, and ICU40) were cured successfully at 1,280 µg/ml sub-inhibitory concentration of acridine orange (Table 7). Isolate ICU40 did not show any change in the resistance profile, while Ab19 and ICU29 showed less resistance to a number of antibiotics. Still, the difference between the cured and non-cured cells did not change the resistance phenotype classification from resistant to sensitive for any of the tested antibiotics. Cured Ab19 was less resistant to amikacin, gentamicin, and meropenem, and its resistance to tobramycin became intermediate.

**Table 5:** Identified plasmids in each isolate, their sizes, characteristics, and resistance genes.

**Isolate**	**Plasmid names**	**Plasmid size**	**Replication group**	**Conjugation genes**	**Resistance genes**
**Ab19**	pAb19.1v1 pAb19.2 pAb19.3	70,616 bp 32,796 bp 6,155 bp	GR6 No rep No rep	Yes No No	*aph(3′)-VIa* *sul2* *ant(2′)-Ia*
**Ab40**	pAb19.1v2	68,848 bp	GR6	Yes	–
**Ab119**	pAb19.1v3	59,004 bp	GR6	Yes	*aph(3′)-VIa*
**ICU29**	pAb-ICU29	8858 bp	GR2	No	–
**ICU40**	pAb19.1v4 pAb-ICU29	70,183 bp 8,858 bp	GR6 GR2	Yes No	*aph(3′)-VIa* -

### Phylogenetic and comparative analysis

**Figure 4:**
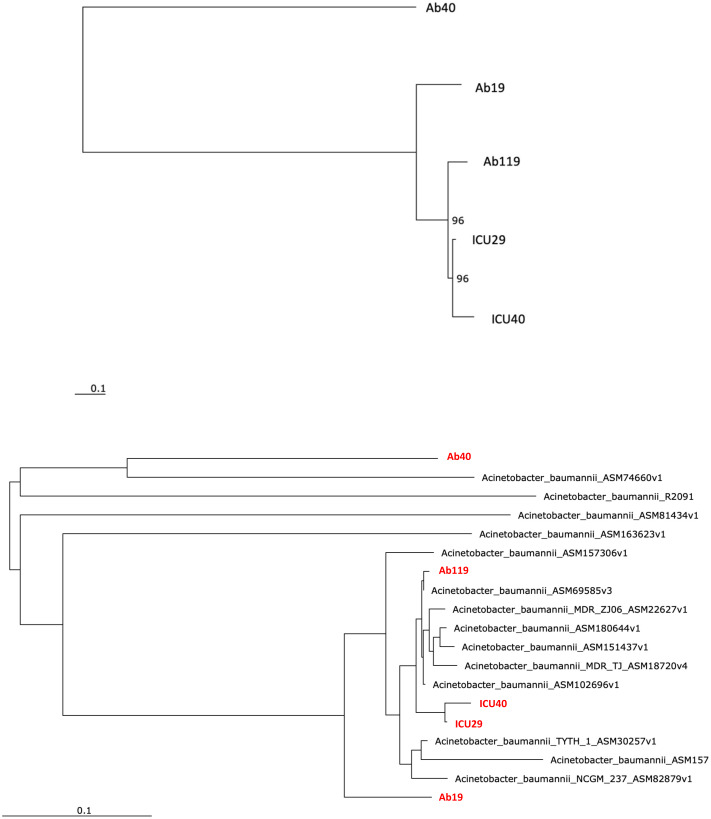
Phylogenetic analysis. (A) Phylogenetic analysis of the five isolates (B) Phylogenetic analysis of the five isolates and with other *Acinetobacter baumannii* strains. Performed using RaXML through EDGE bioinformatics.

Phylogenetic analysis was performed to examine the evolutionary relations between the five isolates (Fig. 4A) and other global *A. baumannii* strains (Fig. 4B). Isolates ICU29 and ICU40 were highly similar, with an ANI of 99.9%, belonged to the same sequence type of the Pasteur scheme, and contained the same plasmid (pAb-ICU29). The two non-ICU isolates, Ab119 and Ab19, showed considerable similarity (ANI: 99.21%). Also, Ab119, Ab19, and ICU40 have a shared plasmid (pAb19.1). Ab40 was the most distant on the phylogenetic tree compared to the other four isolates; ANI between Ab40 and ICU40 was 98.39%.

## Discussion

In Jordan, a recent study on 86 clinical *A. baumannii* isolates revealed that 84.80% of the isolates are XDR (Ababneh et al., 2021). In addition, XDR *A. baumannii* strains were isolated from the surfaces of ICUs and emergency units in major Jordanian hospitals (Ababneh, Abulaila & Jaradat, 2021). Not only clinical isolates, but another study from Jordan showed that toilets and sinks were contaminated with *A. baumannii*, and most of the isolated *A. baumannii* were strong biofilm former (Ababneh, Abu Laila & Jaradat, 2022). Another study detected XDR *A. baumannii* from fruits and vegetables in Jordan (Ababneh, Al-Rousan & Jaradat, 2022).

**Table 6:** Comparison of the resistance profiles of the transconjugant and recipient strains.

**Antibiotic**	**Zone of inhibition in mm (resistance phenotype)**			
	**Recipient strain Pk1**	**Transconjugant strains**		
		**TAb19**	**TAb40**	**TAb119**
Amikacin	22 S	10 R	17 S	19 S
Piperacillin/Tazobactam	30 S	28 S	30 S	30 S
Gentamicin	22 S	22 S	15 I	13 R
Tobramycin	20 S	20 S	20 S	20 S
Ceftazidime	25 S	20 S	ND	19 S
Cefepime	30 S	25 S	11 R	10 R
Ampicillin/Sulbactam	30 S	30 S	34 S	30 S
Doripenem	28 S	30 S	26 S	25 S
Levofloxacin	31 S	30 S	25 S	25 S
Meropenem	35 S	31 S	30 S	31 S
Imipenem	35 S	35 S	35 S	35 S
Ciprofloxacin	30 S	28 S	22 S	23 S
Tetracycline	15 S	13 I	8 R	8 R
Trimethoprim/Sulphamethoxazole	18 S	17 S	17 S	18 S

**Notes.**

R Resistance S Sensitive I Intermediate

**Table 7:** Comparison of the resistance profile of the cured and non-cured strains.

**Antibiotic**	**Ab19**		**ICU29**		**ICU40**	
	**non-cured**	**cured**	**non-cured**	**cured**	**non-cured**	**cured**
Amikacin	0 R	14 R	0	0	0	0
Piperacillin/Tazobactam	0	0	0	0	0	0
Gentamicin	0 R	12 R	0	0	0	0
Tobramycin	0 R	13 I	0	0	0	0
Ceftazidime	0	0	0	0	0	0
Cefepime	0	0	0	0	0	0
Ampicillin/Sulbactam	9	10	11 R	14 I	0	0
Doripenem	8	8	0	0	0	0
Levofloxacin	0	0	0	0	0	0
Meropenem	8 R	12 R	0	0	0	0
Imipenem	8	8	8	8	0	0
Tetracycline	8	8	0	0	0	0
Trimethoprim/Sulphamethoxazole	0	0	0 R	10 R	10	10

**Notes.**

R Resistance S Sensitive I Intermediate

Numbers represent diameters of zones of inhibition.

This study examined and analyzed the WGS of five XDR *A. baumannii* isolates obtained from three major Jordanian hospitals. We focused on characterizing the plasmids and their role in spreading resistance by conjugation. WGS of the XDR isolates investigated in this study revealed many chromosomal and plasmid-borne resistance genes. Consistent with the resistance phenotypes, isolate ICU40 has the highest number of resistance determinants, whereas isolate Ab119 has the least. However, despite this variation in the number of resistance genes, all isolates have very similar resistance profiles, and use various resistance mechanisms. Plasmid-borne resistance was limited to four genes only; two aminoglycoside resistant genes (*aph(3′)-VLa, and ant(2′)-Ia*,) carried by three isolates, and a sulfonamide resistance gene *sul2* on plasmid pAb19.2 of isolate Ab19. Although the *bla*_*OXA*−23_ is known to be harbored on plasmids, it was located on the chromosomal DNA in our isolates (Hamidian et al., 2014b). Also, previous studies have reported the presence of the region “*bla*_*oxa*−23_- ΔATPase” on different types of transposons, mostly Tn2009, Tn2008, Tn2007, and Tn2006. But in our case, the region “*bla*_*oxa*−23_- ΔATPase” was on chromosomal DNA, and none of the previous transposons was present (Chen et al., 2017; Lee et al., 2013).

Although the bacterial isolates were collected from different Jordanian hospitals in different cities, four plasmids out of eight had a shared backbone, while other two plasmids were identical. These similarities indicate that plasmid pAb19.1 may be distributed with minor differences in *A. baumannii* isolates in Jordanian hospitals. In the two ICU isolates, not only was a shared plasmid detected, but they also had a very similar phenotypic resistance profile, virulence genes and ST types. Also, phylogenetic analysis showed that they are highly related, with an ANI of 99.9%. It is noteworth mentioning that ICU29 was isolated from a sputum sample of an 84-year-old female patient, while ICU40 was isolated from the cerebrospinal fluid (CSF) of a 5-week-old male baby in a different hospital located in a different city. However, unlike ICU29, ICU40 carries the novel *bla*_*NDM*−1_ gene encoding the enzyme NDM-1, which confers resistance to *β*-lactam drugs. Moreover, isolate ICU40 carries another plasmid, the pAb19.1v4. The similar ST type, high average nucleotide identity, and the shared plasmid suggest that the two ICU isolates were the same, but later isolate ICU40 acquired the *bla*_*NDM*−1_ gene and the conjugative plasmid pAb19.1. This suggests the existence of a common *A. baumannii* in different ICU wards in Jordanian hospitals. Patients sometimes visit different hospitals and might be transferred later to another hospital in the quest for a cure of a disease that necessitates hospitalization. This explanation is backed by results from a study performed on clinical *A. baumannii* strain that caused an outbreak in Kuwait (Wibberg et al., 2018). The phylogenetic analysis and sequence types were used to link the Kuwait strain (K50) with another *A. baumannii* strain (AA-014) from Iraq that lacks a plasmid found in K50. The authors of this study suggested that both isolates were the same, but horizontal gene transfer events resulted in the additional resistance genes in K50, creating a minor difference between the two isolates.

Most of the recovered plasmids in our study showed similarities with other well-known *A. baumannii* plasmids. For example, plasmid pAb19.3 is highly similar to pRAY*, which is frequently reported in different areas around the world and was not only isolated from *A. baumannii,* but it was also detected in a clinical *Acinetobacter nosocomialis* strain in 2014 (Gifford et al., 2014). Neither pRAY* nor pAb19.3 contains a replication initiation protein. Also, pAb19.1 and its variants detected in four isolates are very similar to pAb-G7-2, which was first isolated from *A. baumannii* GC1 isolate from an Australian hospital and belongs to GR6 like pAb19.1 (Hamidian et al., 2014a). The aminoglycoside resistance transposon TnaphA6 of pAb19.1, which consists of the aminoglycoside resistance gene *surrounded* by two IS*Aba125*, was reported in many plasmids in *A. baumannii* like pAb-G7-2 and pACICU2 (Hamidian & Hall, 2014). One plasmid seems to be novel; pAb19.2 recovered from Ab19 and carries *sul2* resistance gene. A major part of this plasmid (86%) has similarities with pORAB01-2, while the other part did not show similarities with any *A. baumannii* plasmid.

Conjugation experiments generated transconjugants from isolates Ab19, Ab40, and Ab119. The three isolates have a common plasmid pAb19.1 which carries the replication gene *repAci6* and belongs to Group 6 of plasmids (GR6). This group of plasmids have been shown to have the ability to self-transfer (Bertini et al., 2010; Wibberg et al., 2018; Towner et al., 2011; Hamidian et al., 2014b). For example, Leungtongkam et al. (2018) tested the ability of different groups of *A. baumannii* plasmids to self-transfer and found that transconjugants were only obtained from GR6 plasmids  (Leungtongkam et al., 2018). Interestingly, plasmid curing lowered the resistance levels against some antibiotics but did not change the phenotype classification from resistant to sensitive to any of the tested antibiotics. pAb19.1 carrying an aminoglycoside resistance gene may explain the decline in amikacin, gentamicin, and tobramycin resistance. Redundancy in the resistance genes located on the chromosome or the incomplete plasmid curing (Trevors, 1986) may explain why the resistance phenotype didn’t change after the plasmid curing.

Phylogenetic analysis showed that the five isolates are highly similar, especially the two ICU isolates with an ANI of 99.9%. The isolates of this study are similar to clinical strains isolated from various geographical places. For instance, Ab40 is genetically close to *A. baumannii* strain AB031 (ASM74660v1), with an ANI equal to 97.95%. AB031 is a clinical strain that was obtained from a bloodstream infection in a 55-old-patient in Canada (Loewen et al., 2014). The two strains have different sequence types (ST), but similar to AB031, Ab40 uniquely carries the gene *hrgA*, which was identified in many *Helicobacter pylori* strains as it replaced the *hyp* IIIR restriction component (Yakkala et al., 2019). This gene exists in nearly a third of *H. pylori* strains and is more prevalent in strains from gastric cancer patients. Gene *hrgA* was identified in only three *A. baumannii* strains before Ab40 (DS002 Yakkala et al., 2019, AB031 Loewen et al., 2014, AB1297 Hu et al., 2007). In a study on *A. baumannii* strain DS002, Yakkala et al. (2019) suggested that the *hrgA* gene may be transferred to *A. baumannii* from other bacteria by horizontal gene transfer, most probably from *H. pylori*, as a result of co-infecting the same host (Yakkala et al., 2019). Isolate Ab119 have ANI of 99.45% with strain XH857 (ASM157306v1) isolated in China from a sputum sample.

## Conclusions

In the Jordanian *A. baumannii* isolates investigated in this study, many resistance genes are plasmid-borne, and only plasmids of Group 6 showed potential to self-transfer, which emphasizes the role and importance of this group of plasmids in accelerating resistance development among *A. baumannii* clinical strains. In addition, the high similarities between the isolates of this study, especially the two ICU isolates, suggest that there is a common *A. baumannii* strain prevailing in different ICU wards in Jordanian hospitals. Thus, we recommend using the new sequencing technologies instead of the traditional techniques to track the emergence of resistant strains. In addition, plasmid profiling should be expanded to include more isolates, and isolates carrying GR6 plasmids should be further studied because of their increased ability to spread resistance by conjugation.

##  Supplemental Information

10.7717/peerj.14709/supp-1 Supplemental Information 1 Supplemental Tables

## Additional Information and Declarations

### Data Availability

The following information was supplied regarding data availability: The genome sequences of the isolates are available at GenBank: PRJNA739752; GCA_019891135.1, GCA_019891055.1, GCA_019891085.1, GCA_019891095.1, and GCA_019891065.1.
