# Supplementary material for: Extensively drug-resistant Acinetobacter baumannii: role of conjugative plasmids in transferring resistance
Source: PeerJ. 2023 Jan 25;11:e14709. doi: 10.7717/peerj.14709 (PMC9884047; doi:10.7717/peerj.14709)
Supplement: Supplemental Information 1 [file peerj-11-14709-s001.docx]

**Supplementary Data:**

**Table S1.** Antibiotic discs used in Antibiotic Susceptibly Test (AST) according to antibiotic families

| **Antibiotic family** | **Antibiotic** | **Abbreviation** | **Concentration (mcg)** |
| --- | --- | --- | --- |
| **Tetracycline** | Tigecycline | TGC | 15 |
|  | Tetracycline | TE | 30 |
| **Folate pathway antagonists** | Trimethoprim-Sulphamethoxazole | SXT | 1.25/23.75 |
| **β-Lactam combination agents** | Piperacillin/ Tazobactam | TZP | 10 |
|  | Ampicillin-Sulbactam | SAM | 10 |
|  | Ampicillin | AMP | 10 |
|  | Ceftriaxone | CRO | 30 |
|  | Ceftazidime | CAZ | 30 |
|  | Cefepime | FEP | 30 |
| **Carbapenems** | Ertapenem | ETP | 10 |
|  | Doripenem | DOR | 10 |
|  | Imipenem | IPM | 10 |
|  | Meropenem | MEM | 10 |
| **Fluoroquinolones** | Ciprofloxacin | CIP | 5 |
|  | Levofloxacin | LEV | 5 |
|  | Norfloxacin | NOR | 10 |
| **Aminoglycosides** | Gentamicin | CN | 10 |
|  | Amikacin | AK | 30 |
|  | Tobramycin | TOB | 10 |
| **Lipopeptides** | Colistin Sulphate | CT | 10 |
|  | Polymyxin B | Poly B | 10 |

**Table S2.** Overview of the results generated from the assembly.

| Isolates | Ab19 | Ab40 | Ab119 | ICU29 | ICU40 |
| --- | --- | --- | --- | --- | --- |
| Number of contigs | 516 | 598 | 23 | 366 | 888 |
| N50^a^ | 76,198 | 93,333 | 205,931 | 206,661 | 55,820 |
| Maximum contig size (bp) | 276,478 | 255,952 | 537,165 | 560,774 | 159,750 |
| Minimum contig size in bp | 203 | 200 | 6,026 | 204 | 215 |
| Total assembly size in bp | 4,597,552 | 4,485,753 | 3,704,567 | 4,076,889 | 4,712,478 |
| GC content | 38.76% | 38.75% | 38.46% | 38.88% | 38.66% |
| Number of coding sequences | 4,298 | 4,187 | 3,470 | 3,754 | 4,324 |
| Number of Hypothetical coding sequences | 2136 | 2083 | 1625 | 1692 | 2,173 |

a: half the length of the sequence in bp

**Table S3.** Multiple locus sequence typing (MLST) of the isolates according to Pasteur Institute scheme

| **Isolates** | **^Pas^cpn60** | **^Pas^fusA** | **^pas^gltA** | **^Pas^pyrG** | **^Pas^recA** | **^Pas^rplB** | **^Pas^rpoB** | **ST** |
| --- | --- | --- | --- | --- | --- | --- | --- | --- |
| **Ab19** | 2 | 2 | 2 | 2 | 2 | 2 | 2 | 2 |
| **Ab40** | 40 | 3 | 7 | 2 | 40 | 4 | 4 | 164 |
| **Ab119** | 2 | 2 | - | 2 | 2 | 2 | 2 | ND |
| **ICU29** | 2 | 2 | 2 | 2 | 2 | 2 | 1 | 600 |
| **ICU40** | 2 | 2 | 2 | 2 | 2 | 2 | 1 | 600 |

| **Table S4.** Multiple locus sequence typing (MLST) of the isolates according to and Oxford scheme | | | | | | | | |
| --- | --- | --- | --- | --- | --- | --- | --- | --- |
| Isolates | ^Oxf^ gltA | ^Oxf^gyrB | ^Oxf^gdhB | ^Oxf^recA | ^Oxf^cpn60 | ^Oxf^gpi | ^Oxf^rpoD | ST |
| Ab19 | 1 | 12 | 3 | 2 | 2 | 110, 79 | 3 | 1114, 452 |
| Ab40 | 21 | 48 | 58 | 42 | 36 | 114 | 4 | 1418 |
| Ab119 | 3 | 189 | 3 | 2 | 2 | 96 | 191 | New ST |
| ICU29 | 1 | 12 | 189 | 2 | 2 | 193, 96 | 3 | 1305, 2026 |
| ICU40 | 1 | 12 | 189, 3 | 2 | 2 | 195, 114 | 3 | 1632 |
